# Effect of Polymer Concentration and Surface Charge on Controllable Nanopesticides Delivery

**DOI:** 10.3390/polym18131557

**Published:** 2026-06-23

**Authors:** Ran Cao, Yue Wu, Nuo Xu, Yutao Zhang, Zhiqian Guo, Yisheng Xu

**Affiliations:** 1 State Key Laboratory of Green Chemical Engineering and Industrial Catalysis, School of Chemical Engineering, East China University of Science and Technology, Shanghai 200237, China; 2 Guangxi Laboratory of Low-Carbon Technology and Green Chemical Advanced Materials, Qinzhou 535008, China; 3 State Key Laboratory of Chemical Engineering and Low-Carbon Technology, School of Chemical Engineering, East China University of Science and Technology, Shanghai 200237, China; 4 Key Laboratory for Advanced Materials, East China University of Science and Technology, Shanghai 200237, China; 5 MOE Joint International Research Laboratory of Precision Chemistry and Molecular Engineering, East China University of Science and Technology, Shanghai 200237, China; 6 Feringa Nobel Prize Scientist Joint Research Center, Institute of Fine Chemicals, School of Chemistry and Molecular Engineering, East China University of Science and Technology, Shanghai 200237, China

**Keywords:** nanopesticides, NIR-II fluorescence, flash nanoprecipitation, surface charge

## Abstract

The efficacy of polymer-based nanopesticides (NPs) is strongly governed by carrier concentration and surface charge, which affect shell thickness, drug release kinetics, and photostability. However, the influence of these two factors in pesticide release and delivery performance remains unclear. This study introduces a NIR-II fluorescence dye-tracing strategy to enable high-resolution monitoring of NP behavior in model plants. By systematically varying polymer concentration and copolymer blocks, we investigate their impact on release behavior, photostability, and stem uptake. As the polymer concentration increased, NPs demonstrated a controlled slow release and better photostability, yet a lower pesticide loading capability. In model plants, PISNPs transport quickly and can accumulate at wound sites, effectively offering antifungal properties. This work provides experimental evidence for optimizing polymer carrier design to achieve efficient, controlled release while minimizing photodegradation risks, offering practical guidelines for developing high-performance, low-risk nanopesticide formulations.

## 1. Introduction

The rapid development of nanotechnology has provided new strategies for plant protection. Among these, nanopesticides have become a hot point in agricultural nanotechnology research due to their unique size effects and carrier delivery properties [1,2,3,4]. Although nanopesticides demonstrate significant advantages in enhancing efficacy and reducing toxicity, their functionality is highly dependent on the physicochemical properties of the carrier materials [5,6,7,8,9]. In particular, the concentration of polymer carriers and the surface charge induced are key factors determining the release behavior and stability of nanopesticides. Existing research indicates that polymer concentration directly influences the shell thickness, crosslinking density, and encapsulation efficiency of nanocarriers [10,11,12]. Meanwhile, the surface charge introduced by polymer blocks of different structures regulates the diffusion rate of drug molecules from the interior of the carrier to the external environment, thereby affecting their release behaviors [13,14]. However, there is currently a lack of systematic and clear understanding regarding how polymer concentration and polymer blocks synergistically influence the release behavior of nanopesticides within plants, as well as the mechanisms by which these factors affect photostability.

To thoroughly elucidate the distribution patterns and dynamic behavior of nanopesticides, there is an urgent need to establish highly sensitive, visual in situ tracking methods. In recent years, fluorescent dye tracing technology has been widely applied in studies of the biological distribution of nanocarriers due to its advantages of ease of operation, high spatiotemporal resolution, and good biocompatibility [15,16,17,18,19]. By co-encapsulating or chemically bonding fluorescent dyes with pesticide molecules within nanocarriers, the accumulation and transport processes of nanoparticles in different plant tissues can be directly observed using laser confocal microscopy, or in vivo imaging systems [20,21,22,23,24,25,26].

**Figure 1:**
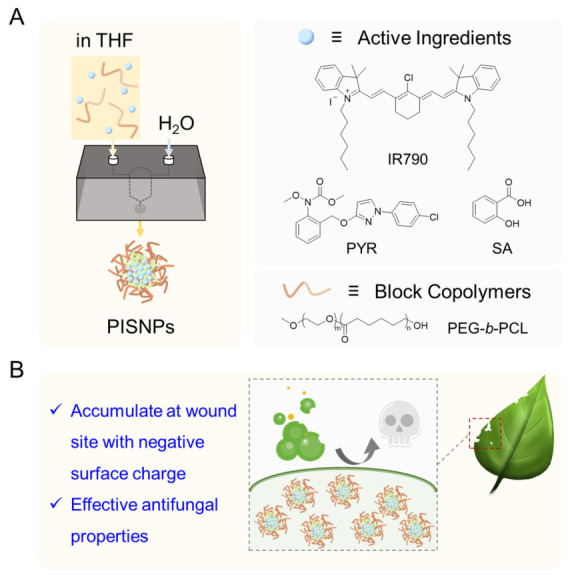
(**A**) Preparation of PISNPs (**B**) Controllable nanopesticides delivery.

Herein, this study aims to incorporate fluorescent dyes into nanopesticide delivery systems to systematically investigate the tissue distribution, release kinetics, and photostability of nano-pesticides in model plants under varying polymer concentrations and surface charge conditions. The flash nanoprecipitation (FNP) method was employed to co-encapsulate both pyraclostrobin (PYR) pesticide and NIR-II fluorescent dye (IR790) using amphiphilic block copolymers, introducing salicylic acid (SA) as a counterion to locally modulate the aggregation state of IR790 within one nanopesticide system (PISNPs) (Figure 1A). Furthermore, by systematically varying the polymer concentration and copolymer block compositions, positively or negatively charged nanocarriers were formulated to achieve controlled release and tunable plant transport behavior (Figure 1B). Subsequently, due to the dense polymer shell formed at higher polymer concentrations, the release of pesticide was sustained and photostability was enhanced, acting as a physical barrier that prevents rapid photodegradation. Moreover, the antifungal properties and the real-time transport behavior of PISNPs in cut-flower models were systematically investigated using NIR-II fluorescence imaging. This strategy of combining tunable polymer concentration, copolymer block design, and NIR-II dye tracing offers a novel platform for nanopesticide engineering, which realizes a breakthrough for in situ monitoring of pesticide distribution and sustained release, supporting precision plant protection with reduced biotoxicity.

## 2. Materials and Methods

### 2.1. Materials

Pyraclostrobin (PYR, 98%) and THF were purchased from Shanghai Adamas beta Co. Salicylic acid (SA, 98%) was obtained from Shanghai Bidepharm Co. (Shanghai, China) IR790 was synthesized according to previous report [27]. Poly-(2-(dimethylamino) ethylmethacrylate)-b-poly(ε-caprolactone) (Relative Molecular Mass: 4k-*b*-10k, PDMA-*b*-PCL) was synthesized according to previously reported work [15]. PEG-*b*-PCL (Relative Molecular Mass: 5k–10k) was purchased from Shanghai Yanyi Biological Co. (Shanghai, China). Dialysis membrane was purchased from Shanghai Yuanye Biotechnology Co. (Shanghai, China). All the reagents, which were not especially pointed out, were analytical grade, and ultra-pure water was used in all experiments. Ultra-pure water was obtained by a Milli-Q water purification system.

### 2.2. Characterization

The hydrodynamic diameter, polydispersity index (PDI), and zeta potential of nanoparticles were measured using a dynamic light scattering instrument (Malvern Zetasizer Pro Blue, ZSU3200, Malvern Panalytical, Malvern, UK). Prior to each measurement, the sample was equilibrated at 25 °C for 120 s to ensure thermal stability, and each sample was measured at least three times. The stability of nanoparticles was evaluated over the vase period by monitoring changes in particle size under room temperature. The morphology of nanoparticles was characterized by scanning transmission electron microscopy (STEM) using a Talos F200X transmission electron microscope (Thermo Fisher Scientific, Waltham, MA, USA). For STEM imaging, 20 μL of nanoparticle suspension was deposited onto a 200-mesh carbon-coated copper grid, allowed to adsorb for 1 h at room temperature, and then air-dried; images were acquired in STEM mode at an accelerating voltage of 30 kV. UV–vis absorption spectra were recorded over 200–900 nm using a UV–vis spectrophotometer (Agilent, Santa Clara, CA, USA) with ultrapure water as reference. Fluorescence emission spectra were recorded from 810 to 1000 nm with an excitation wavelength of 790 nm. Fourier transform infrared (FT-IR) spectra were recorded using a Spectrum 100 FTIR spectrometer (Perkin Elmer Inc., Shelton, CT, USA) with 32 scans over a wavenumber range of 500–4000 cm^−1^.

### 2.3. Synthesis of PISNPs by FNP

PISNPs were prepared by flash nanoprecipitation (FNP) using a confined impinging jet with dilution (CIJ-D) mixer. Briefly, polymeric materials including PEG-*b*-PCL and PDMA-*b*-PCL were dissolved in THF together with IR790, SA, and PYR. The organic phase was rapidly mixed with an equal volume of aqueous phase under controlled flow rates using a syringe pump, followed by dilution in ultrapure water. Under optimized conditions, the concentrations were set as follows: IR790 at 8 μM; SA at 80 μM; PYR at 0.1 mg mL^−1^; total polymer concentration, 0.5 mg mL^−1^; and flow rate at 30 mL min^−1^. The resulting nanoparticles were further purified by dialysis to remove free molecules and residual solvent. After the FNP process, the obtained nanoparticle suspension was transferred into a dialysis bag (molecular weight cutoff: 3500 Da) and dialyzed against ultrapure water for 12–16 h to remove organic solvent and unencapsulated small molecules (free IR790, SA, and PYR). After dialysis, the fluorescent nanoparticle solution was stored in the dark at room temperature prior to use.

### 2.4. Encapsulation Efficiency and Drug Loading Capacity

The amount of encapsulated PYR was determined by UV-vis spectrophotometry. Specifically, the absorbance of PYR at its characteristic peak (ca. 275 nm) was measured in the nanoparticle dispersion after purification by dialysis (using a 3500 Da dialysis bag against ultrapure water for 12–16 h). The concentration of encapsulated PYR was calculated using a standard calibration curve prepared from PYR solutions of known concentrations. EE and DLC were then calculated according to the following equations:

(1) $$EE\left(\%\right)=\frac{amount\;of\;encapsulated\;PYR}{total\;amount\;of\;PYR\;added}\times 100\%$$

(2) $$DLC\left(\%\right)=\frac{amount\;of\;encapsulated\;PYR}{total\;mass\;of\;nanoparticles}\times 100\%$$

### 2.5. Photostability Measurement

The photostability of PISNPs was evaluated under UV irradiation. PISNP dispersions prepared with different polymer concentrations (0.02–1.0 mg/mL) or different PDMA block ratios (0–100 wt%) were diluted to the same PYR concentration. A 5 mL aliquot of each dispersion was placed in a quartz cell (diameter: 60 mm) and irradiated in a dark cabinet equipped with a UV lamp (254 nm, 16.4 W/m^2^, ~10 cm from the sample). At predetermined time intervals (0.5, 1, 2, 4, 6, 8, and 12 h), an aliquot was withdrawn, diluted with THF, and analyzed by UV-vis spectroscopy (absorbance at ca. 275 nm). The cumulative remaining ratio was calculated as

(3) $$Cumulative\;remaining\;ratio(\%)=\frac{A_{t}}{A_{0}}\times 100\%$$

where $A_{0}$ and $A_{t}$ are the initial absorbance and the absorbance at time t, respectively. All experiments were performed in triplicate.

### 2.6. Transport of PISNPs in Cut Stems

Cut flowers (*Rosa hybrida*, ‘Carola’) were purchased from a local flower market in Shanghai, China. Stem segments (5 cm) were cut at a 45° angle and longitudinally sectioned. The samples were immediately immersed in 1 mL nanoparticle dispersions in a quartz cuvette. NIR-II fluorescence imaging was performed to monitor nanoparticle transport in real time over 0–60 s using NIR-II in-vivo imaging system (Artemis Intelligent Imaging, Shanghai, China, MARS 900–1700 nm), $\lambda_{ex}$ = 808 nm. The transport height was defined as the distance from the liquid level to the highest observable fluorescence signal along the stem. Each experiment was repeated three times.

### 2.7. Statistical Analysis

The experimental data were analyzed using mean ± standard deviation (SD) with at least three experiments (*n* ≥ 3). ANOVA was used to assess statistical significance: * *p* < 0.05; ** *p* < 0.01; *** *p* < 0.001. Origin 2018 software was used for statistical analysis.

## 3. Results and Discussion

PYR/IR790/SA nanopesticides (PISNPs) are prepared by PYR (pesticides), IR790 (NIR-II dye) [27], SA (counterion molecular), and block copolymers via the flash nanoprecipitation (FNP) method. Through the FNP method, different block copolymers and process parameters could be manipulated [28,29,30,31,32,33], which enhances the PISNP fluorescence intensity and improves the pesticide release and photostability. The PISNP particle sizes (Figure S1) and particle sizes stability (Figure S2) obtained at different PYR concentrations are relatively similar, and all exhibited distinct negative charges (Figure S3).

### 3.1. Screening of IR790 and SA Concentrations for PISNP NIR-II Fluorescence Intensity

To enable the accurate visualization of nanoparticle biodistribution in model plant stems, near-infrared II (NIR-II) fluorescent nanopesticides (NPs) were developed and systematically optimized. IR790 was used as the fluorescent probe, and salicylic acid (SA) was introduced to improve encapsulation efficiency.

**Figure 2:**
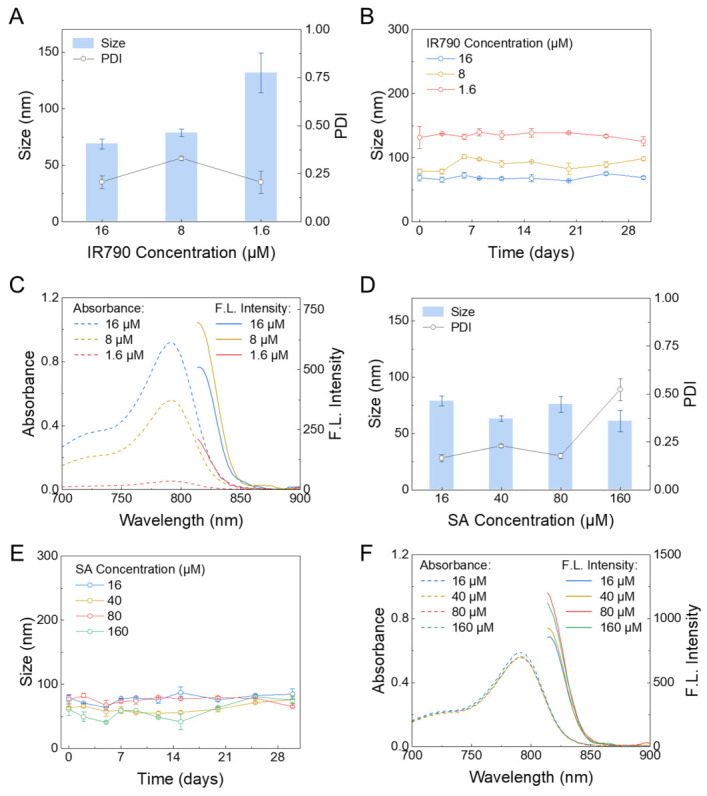
(**A**) Particle size, PDI, and (**B**) size stability of NPs at different IR790 concentrations. (**C**) Absorption spectra and emission spectra of NPs at different IR790 concentrations. (**D**) Particle size, PDI, and (**E**) size stability of NPs at different SA concentrations. (**F**) Absorption spectra and emission spectra of NPs at different SA concentrations.

As shown in Figure 2A, the particle size of the nanoparticles gradually decreased with increasing IR790 concentration, and all particles were negatively charged (Figure S4). The PISNPs also exhibited good size stability at different IR790 concentrations (Figure 2B). Meanwhile, as the IR790 concentration increased, the intensity of the absorption peak increased (Figure 2C). However, under excitation at 790 nm, the fluorescence intensity of the PISNPs first increased and then decreased. This is because, at excessively high dye concentrations, strong interactions resulting from molecular aggregation led to a pronounced aggregation-induced quenching (ACQ) effect, which reduces the fluorescence intensity [34]. In contrast, at 8 μM, the dye concentration and aggregation level reached an optimal balance, yielding higher overall fluorescence intensity.

The introduction of SA is expected to further weaken the aggregation of IR790 molecules, enabling the formation of a more dispersed state of IR790 within the PISNPs. As shown in Figure 2D, the PISNPs obtained at different SA concentrations have a particle size of approximately 70 nm, exhibiting good particle size stability (Figure 2E) and negative zeta potentials (Figure S5). However, the particle size distribution index (PDI) of PISNPs increases to about 0.5 at SA concentration of 160 μM, indicating a significant decline in particle uniformity. These results indicate that at excessively high SA concentrations, SA may potentially compete with IR790, disrupting the original assembly, resulting in poorer dispersion. As shown in Figure 2F, the fluorescence intensity of PISNPs increased significantly upon the addition of SA, reaching a maximum at an SA concentration of 80 μM. However, when SA was further increased to 160 μM, the excess SA may have over-participated in the PISNP assembly, leading to a slight decrease in fluorescence intensity. The optimized formulation parameters were determined as follows: a dye concentration of 8 μM and an SA concentration of 80 μM.

### 3.2. Polymer Concentration Effect on PISNP Release and Photostability

**Figure 3:**
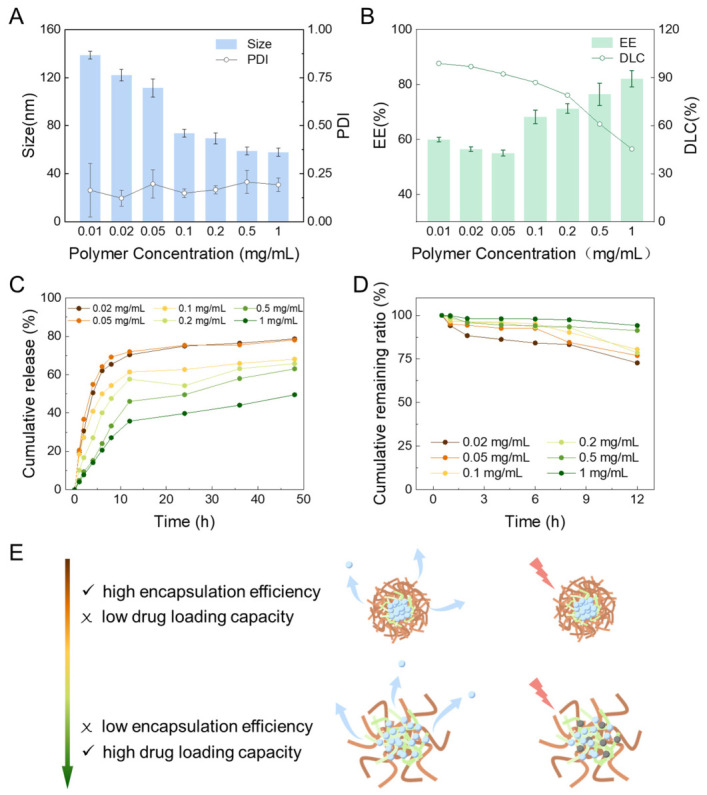
(**A**) Particle size and PDI of PISNPs at different polymers concentrations. (**B**) EE and DLC of PISNPs at different polymers concentrations. (**C**) Pesticide release and (**D**) photostability of PISNPs at different polymers concentrations varying from 0.02 mg/mL to 1 mg/mL. (**E**) Schematic structure of PISNPs at different polymer concentrations.

To investigate the effect of polymer concentration on the pesticide release and photostability performance of PISNPs, the PEG-*b*-PCL concentration was adjusted from 0.01 mg/mL to 1 mg/mL. As shown in Figure 3A, an increase in polymer concentration resulted in a decrease in the particle size of the PISNPs, while PDI remained around 0.2, indicating the excellent dispersibility of the PISNPs. As the polymer concentration increased, the encapsulation efficiency (EE) rose to 80%, while the drug loading capacity (DLC) correspondingly decreased (Figure 3B). The EE was measured by comparison with the standard curve (Figure S6). Moreover, all PISNPs demonstrated negative zeta potential due to the negative PEG-*b*-PCL micelles (Figure S7). Since the stability and dispersibility were poor at the 0.01 mg/mL concentration, this condition was excluded from subsequent testing. This necessitates maintaining a high drug loading capacity while preserving a high encapsulation efficiency. As the polymer concentration increased, the absorption also gradually enhanced, following the same trend as the EE (Figure S8). However, the fluorescence intensity of PISNPs exhibited a pattern of first increasing and then decreasing (Figure S9). This is because, as the polymer concentration rose, more dyes were encapsulated within the PISNPs, which the particle size decreased at this point. Consequently, the aggregation of IR790 increased within a confined environment, leading to a certain degree of fluorescence quenching.

Given the weakly acidic properties of plant tissue in the stem, experiments were conducted to examine the release of PISNPs under the pH = 6.6 condition. As shown in Figure 3C, the release of the pesticide gradually slows with increasing polymer concentration, demonstrating a sustained-release effect. At lower polymer concentrations, the PISNPs is completely released within 12 h, resulting in reduced bioavailability and an inability to achieve the goal of long-lasting antimicrobial activity. This is because a higher polymer concentration produces a thicker, denser shell around the PISNPs, which physically impedes drug diffusion, thereby slowing release. The release kinetics of PYR from PISNPs at different polymer concentrations were analyzed using the Korsmeyer–Peppas model, a widely employed empirical model for describing drug release from nanoparticles [35,36,37]. The fitting results, presented in Figure S10 and Table S1, show that the release data are well described by this model. Similarly, as the polymer concentration decreases, the photostability of PISNPs declines (Figure 3D). This is because a higher polymer concentration results in a denser polymer layer (Figure 3E), which blocks contact between the pesticide and light, thereby slowing the photodegradation of PISNPs.

### 3.3. Impact of Copolymer Blocks on PISNPs Release and Photostability

To investigate the effect of different polymer hydrophobic blocks on the EE of PISNPs, PISNPs were fabricated by PEG-*b*-PLGA (PLGA) and PEG-*b*-PCL (PCL), respectively. Compared to PCL, the PISNPs prepared utilizing the PLGA block performed larger particle sizes (Figure S11), but both PISNPs exhibit good particle stability (Figure S12). Moreover, the EE of PLGA was lower than that of PCL (Figure S13), which is because PCL is more hydrophobic than PLGA, making it easier for PYR to co-precipitate with the hydrophobic PCL block during the FNP process.

**Figure 4:**
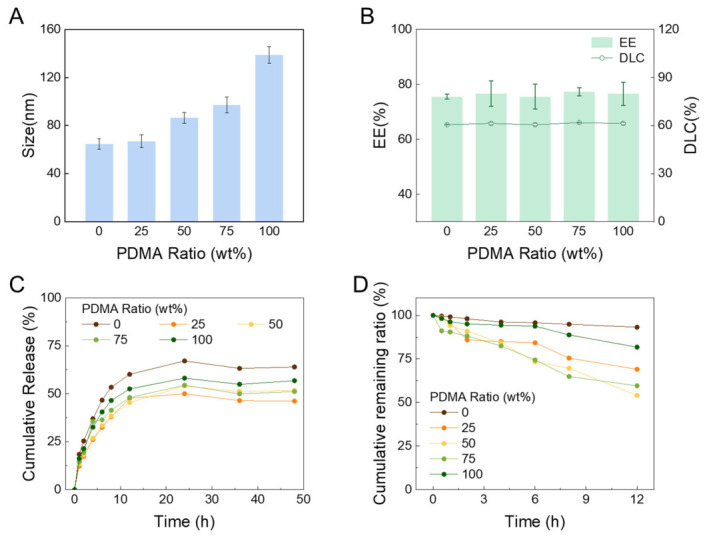
(**A**) Particle size of PISNPs at different PDMA polymer blocks ratio. (**B**) EE and DLC of PISNPs at different PDMA polymer blocks ratio. (**C**) Pesticide release and (**D**) photostability of PISNPs at different PDMA polymer blocks ratio varying from 0 wt% to 100 wt%.

Furthermore, biocompatible block copolymers with different polymer hydrophilic blocks, PDMA-*b*-PCL (PDMA) and PEG-*b*-PCL (PEG), were used as carriers. As shown in Figure 4A, the particle size of PISNPs gradually increased with the rise in the PDMA ratio, maintaining good dispersion. This result is attributed to the fact that as the hydrophilic PEG is gradually replaced by PDMA, the ability of PDMA to stabilize the nanoparticles is weaker, thus requiring more polymer and a longer stabilization time, leading to continuous nanoparticle growth and consequently promoting an increase in particle size [38]. In contrast, the EE and DLC of PISNPs did not change with the polymer block (Figure 4B), which suggested that the hydrophilic block has little effect on EE. As shown in Figure S14, the zeta potential of PISNPs shifts from negative to positive when the PDMA ratio increases. After the introduction of PDMA, the absorption of PISNPs at 790 nm was significantly reduced overall (Figure S15); meanwhile, the fluorescence intensity was significantly decreased as the PDMA ratio increased (Figure S16).

At similar release rates, the main distinction lay in the amount released; the higher the absolute surface charge was, the more pesticides the PISNPs released (Figure 4C). Higher absolute zeta potential enhances electrostatic repulsion between polymer chains, loosening the shell structure and accelerating drug diffusion. The release kinetics of PYR from PISNPs with varying PDMA block ratios were also fitted using the Korsmeyer–Peppas model (Figure S17 and Table S2). The model describes the release behavior well across all formulations. The trends in photostability performance were opposite to those in release performance (Figure 4D). Higher absolute zeta potential prevents nanoparticle aggregation, reducing drug exposure to the external environment, thereby protecting the encapsulated pesticide from photodegradation. Upon alteration of the polymer hydrophilic blocks, the pesticide release and photostability performance were related to the surface charge.

### 3.4. Particle Performance of Optimized PISNPs

**Figure 5:**
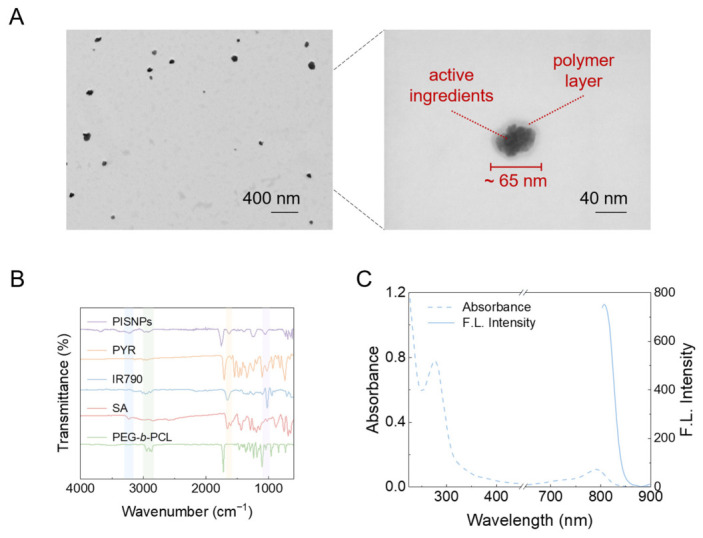
Photophysical properties of optimized PISNPs. (**A**) STEM images of PISNPs. (**B**) FT-IR spectra of PISNPs, PYR, IR790, SA, and PEG-*b*-PCL. (**C**) Absorption spectra and emission spectra of PISNPs ($\lambda_{ex}$ = 790 nm).

To verify the fluorescence intensity of PISNPs in a cut-flower model plant and the accumulation at wound sites, the optimized PISNPs were characterized. As shown in Figure 5A and Figure S18, STEM images reveal that PISNPs were nearly spherical, with a particle size of approximately 65 nm. A distinct light-colored polymer layer and the dark-colored active ingredients inside can be observed. As illustrated in the FT-IR spectrum (Figure 5B), the PISNPs retain the characteristic absorption peaks of all individual components. For PYR, the C=O stretching vibration appears around 1710 cm^−1^. For IR790, the conjugated C=C stretching vibration of the polymethine chain is observed around 1600 cm^−1^, confirming the presence of the NIR-II dye. SA exhibits a broad and diffuse O–H stretching band in the range of 3200–2500 cm^−1^, characteristic of its carboxylic acid group. The PEG-*b*-PCL block copolymer shows strong C–H stretching bands around 2900 cm^−1^, attributed to the long alkyl chains of PCL and the ethylene glycol units of PEG. All these characteristic peaks are clearly identifiable in the PISNPs spectrum, demonstrating the successful co-encapsulation of PYR, IR790, SA, and the polymer carrier within the nanopesticide formulation. As shown in Figure 5C, the absorption and fluorescence spectra further demonstrated that PISNPs have successfully loaded PYR and IR790 and exhibit strong fluorescence intensity.

### 3.5. Antifungal Properties and Transport Behavior of PISNPs

**Figure 6:**
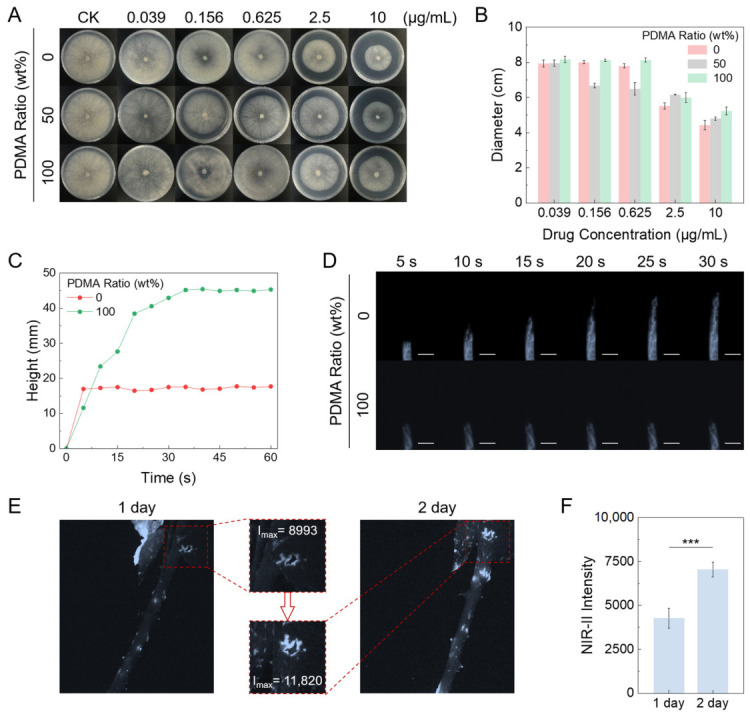
Antifungal properties and transport behavior of PISNPs. (**A**) Images of the fungicidal activity of PISNPs *B. cinerea* after treatment. (**B**) Inhibition rate at different drug concentrations against *B. cinerea*. The concentration of PYR were 0.039, 0.156, 0.625, 2.5, and 10 μg/mL. (**C**) Time-dependent transport height in cut stems (0–60 s) and (**D**) corresponding NIR-II imaging of PISNPs uptake at the first 30 s. Scale bar: 10 mm. (**E**) NIR-II images of cut flower stem and leaves with enlarged wound sites with 5 min PISNPs treatment after 2 days. (**F**) The NIR-II fluorescence intensity of wound sites of leaves with 5 min PISNPs treatment after 2 days. Data are presented as mean ± standard deviation. *p*-values are calculated by using one-way ANOVA with Tukey test, *** *p* < 0.001.

The antifungal properties of PISNPs were tested against *B. cinerea* as indicator organisms. CK served as the control group, indicating high fungal activity and good reproductive capacity. The results are shown in Figure 6A,B; PISNPs prepared under 0 wt% PDMA conditions exhibit good antifungal activity at high concentrations.

NIR-II fluorescence imaging was used to monitor the real-time transport behavior of nanoparticles with different charges within cut flower stems. After the stems were longitudinally incised, they were immediately inserted into cuvettes containing PISNPs. Neither deionized water nor free IR790 suspension produced detectable fluorescence at wound sites (Figure S19). As shown in Figure 6C, PISNPs were able to enter the vascular bundles and move upward along the stems, but their transport behavior exhibited significant charge-dependent differences. Negatively charged PISNPs (0 wt%) exhibited faster transport speeds, with their fluorescence signals rapidly propagating upward along the vascular bundles and reaching a high transport height by 60 s (Figure S20). In contrast, the transport speed of positively charged PISNPs (100 wt%) was markedly reduced; their fluorescence signals were primarily concentrated near the incision site, and the distance of upward transport was significantly limited (Figure 6D). To quantitatively evaluate the effect of surface charge on transport behavior, the transport heights of PISNPs with different PDMA content were compared (Figures S21 and S22). The results show that as the zeta potential became more positive with increasing PDMA content, the transport height progressively decreased, confirming that electrostatic attraction between positively charged nanoparticles and negatively charged plant cell walls restricts upward translocation. To verify accumulation of PISNPs at the wound sites to provide antifungal function, cut flower (*Rosa hybrida*, ‘Carola’) was used to investigate the biodistribution of PISNPs within the plant. Stems were recut at a 45° angle to a length of 35 cm, placed in a solution containing PISNPs for 5 min, and then removed. A NIR-II in vivo imaging system was used to observe the fluorescence intensity in the stem and leaf regions (Figure 6E,F). After 24 h, the fluorescence signal was primarily observed near the cut surface and damaged leaf surfaces; after 2 days, the fluorescence continued to intensify. The in planta application of PISNPs was further investigated (Figure S23). Cut roses pretreated with PISNPs remained largely asymptomatic after 6 days, whereas water-pretreated controls developed extensive lesions, confirming the effective suppression of gray mold development by PISNPs.

## 4. Conclusions

In this work, PYR/IR790/SA nanopesticides (PISNPs) are prepared by the FNP method with the adjustment of block copolymers, realizing the encapsulation of a NIR-II fluorescent dye together with the pesticides. The effects of different polymer concentrations and copolymer block compositions on the pesticide release and photostability of PISNPs are investigated. There is an optimized formulation, where an appropriate polymer concentration leads to a denser polymer layer, causing slower release and enhanced photostability, while negatively charged PISNPs exhibit faster vascular transport and accumulation at wound sites. Additionally, NIR-II fluorescence imaging enables real-time visualization of charge-dependent transport behavior in plant stems, providing ideas for improving the targeted delivery and antifungal efficacy of nanopesticides.

## Data Availability

The original contributions presented in this study are included in the article/Supplementary Materials. Further inquiries can be directed to the corresponding authors.

## Supplementary Materials

The following supporting information can be downloaded at: https://www.mdpi.com/article/10.3390/polym18131557/s1.
