# Supplementary material for: Effect of Polymer Concentration and Surface Charge on Controllable Nanopesticides Delivery
Source: Polymers (Basel). 2026 Jun 23;18(13):1557. doi: 10.3390/polym18131557 (PMC13363951; doi:10.3390/polym18131557)
Supplement: Supplementary file 1 [file polymers-18-01557-s001.zip › polymers-4371958-supplementary.pdf]

polymers

Supplementary Materials

Effect of polymer concentration and surface charge on controllable nanopesticides delivery

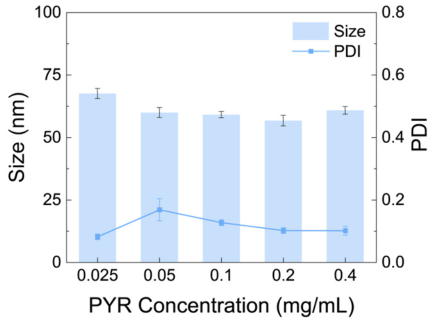

Figure S1. Particle size and PDI of nanoparticles at different PYR concentrations.

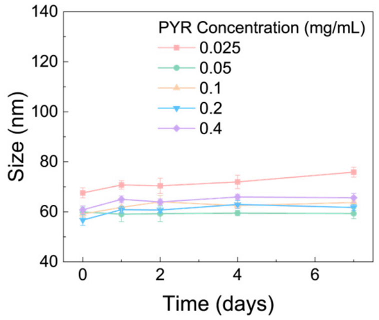

Figure S2. Stability of nanoparticles at different PYR concentrations.

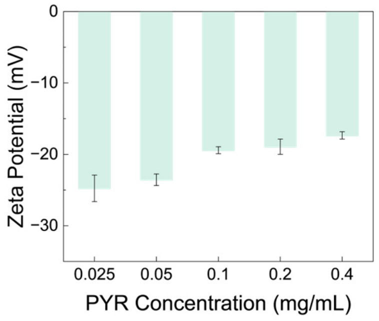

Figure S3. Zeta Potential of nanoparticles at different PYR concentrations.

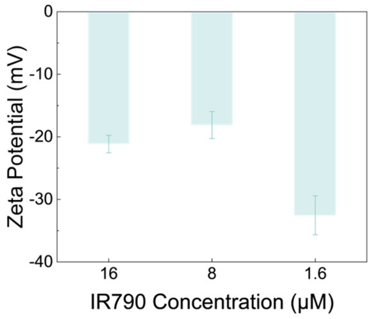

Figure S4. Zeta potential of nanoparticles at different IR790 concentrations.

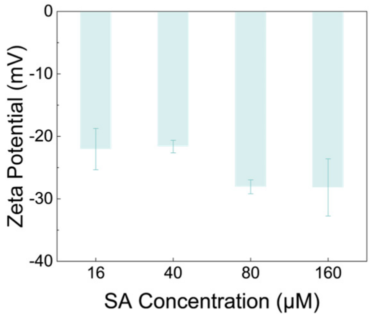

Figure S5. Zeta potential of nanoparticles at different SA concentrations.

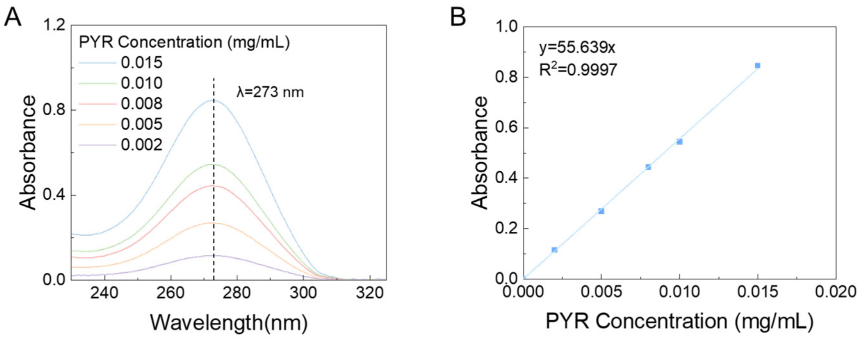

Figure S6. (A) Absorbance spectrum and **(B)** standard calibration curve of PYR with different concentrations.

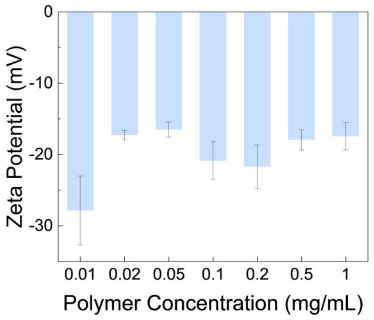

Figure S7. Zeta potential of PISNPs at different polymer concentrations.

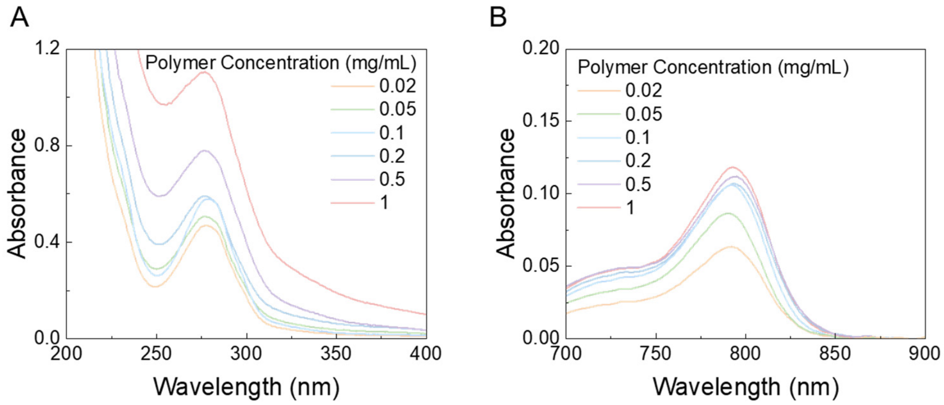

Figure S8. Absorption spectra of PISNPs with different polymer concentrations.

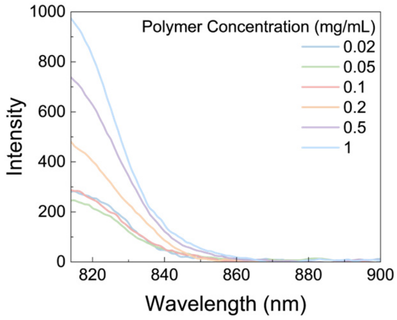

Figure S9. Emission spectra of PISNPs at different polymer concentrations (λ_{ex} = 790 nm).

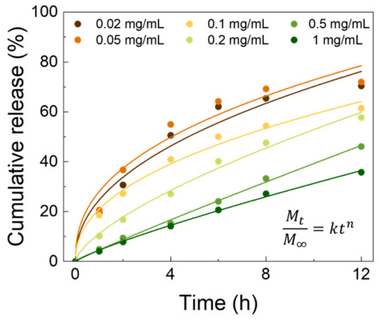

Figure S10. Fitting of the cumulative release data of PYR from PISNPs at different polymer concentrations using the Korsmeyer-Peppas model (M_t/M_∞: cumulative fraction of PYR released at time t; k: release rate constant; n: release exponent).

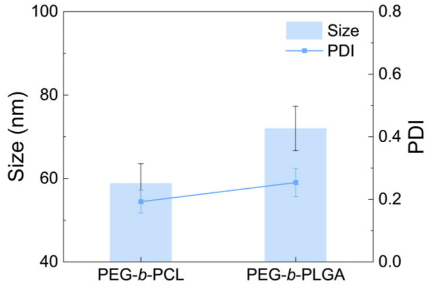

Figure S11. Particle size and PDI of nanoparticles with different polymer types.

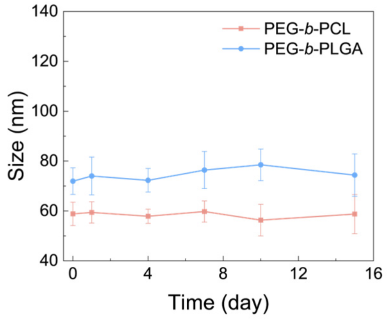

Figure S12. Stability of nanoparticles with different polymer types.

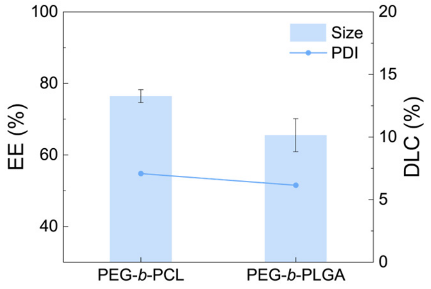

Figure S13. Encapsulation efficiency and drug loading capacity of nanoparticles with different polymer types.

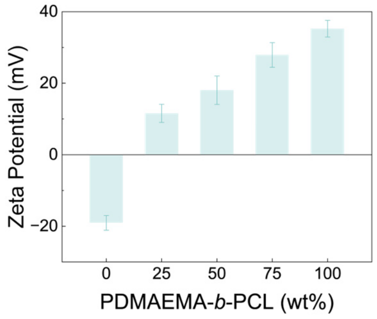

Figure S14. Zeta potential of PISNPs at different PDMA ratio.

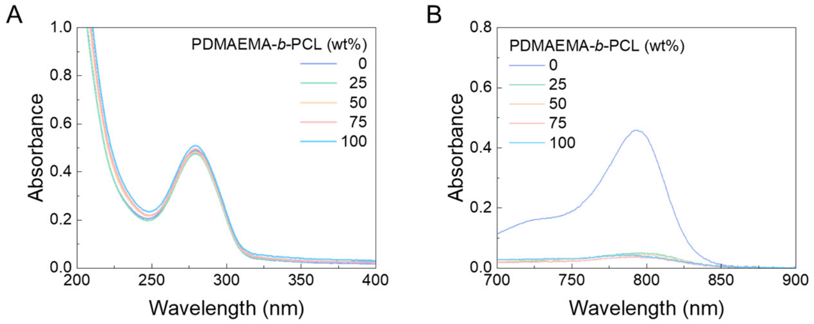

Figure S15. Absorption spectra of PISNPs at different PDMA ratio.

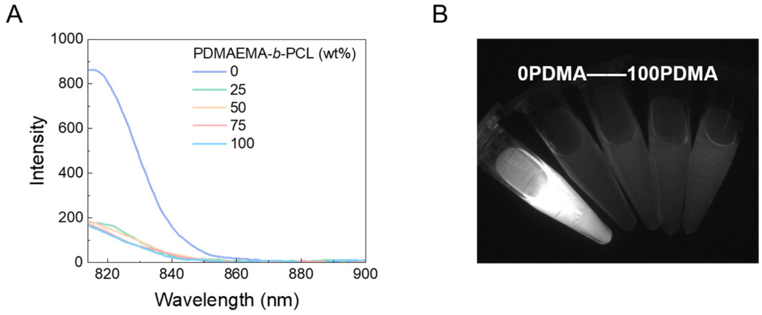

Figure S16. (A) Emission spectra and **(B)** NIR-II fluorescence images of PISNPs at different PDMA ratio (λ_{ex} = 790 nm).

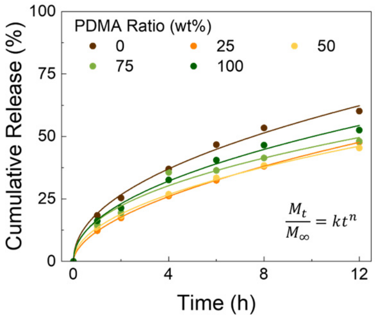

Figure S17. Fitting of the cumulative release data of PYR from PISNPs at different PDMA block ratios using the Korsmeyer-Peppas model (M_t/M_∞: cumulative fraction of PYR released at time t; k: release rate constant; n: release exponent).

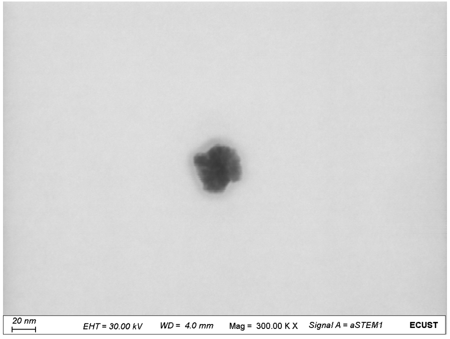

Figure S18. STEM image of PISNPs.

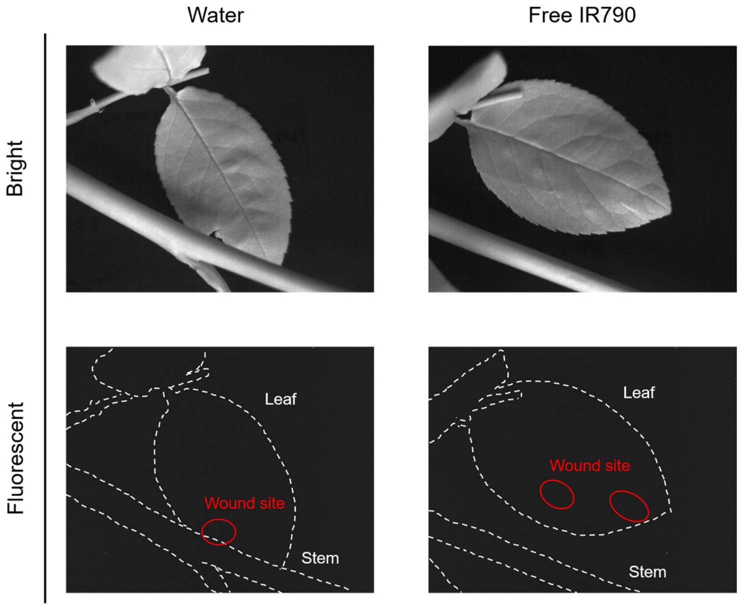

Figure S19. NIR-II images of cut flower stem with 5 min deionized water treatment and free IR790 treatment.

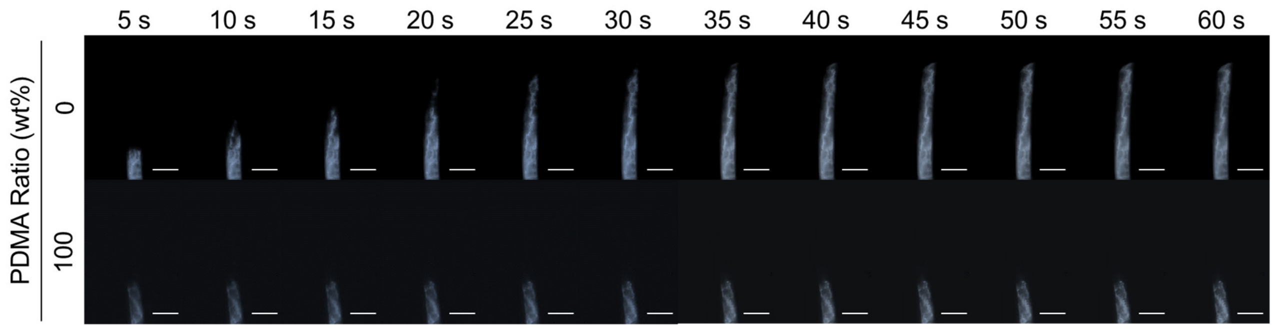

Figure S20. Time-dependent NIR-II fluorescence imaging of nanoparticle transport in stems at different PDMA ratio (λ_{ex} = 790 nm).

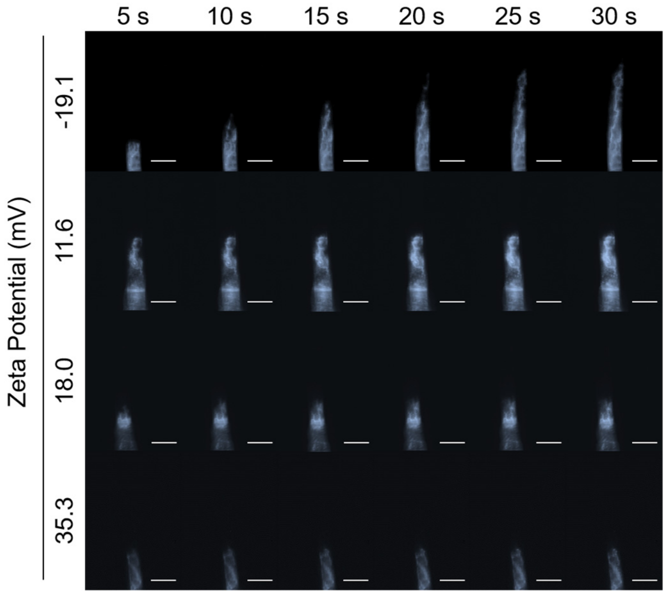

Figure S21. NIR-II fluorescence images of PISNPs uptake with different surface charge in cut rose stems over the first 30 s. Scale bar: 10 mm.

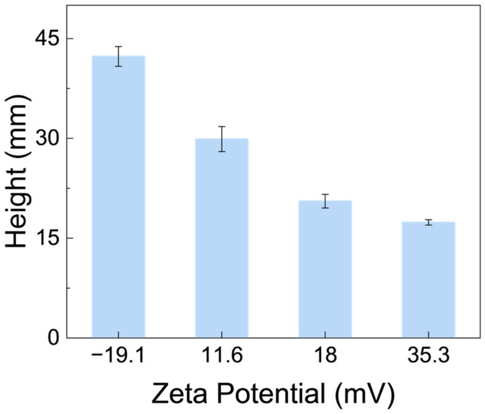

Figure S22. Transport height of PISNPs with different surface charge in cut rose stems at 30 s.

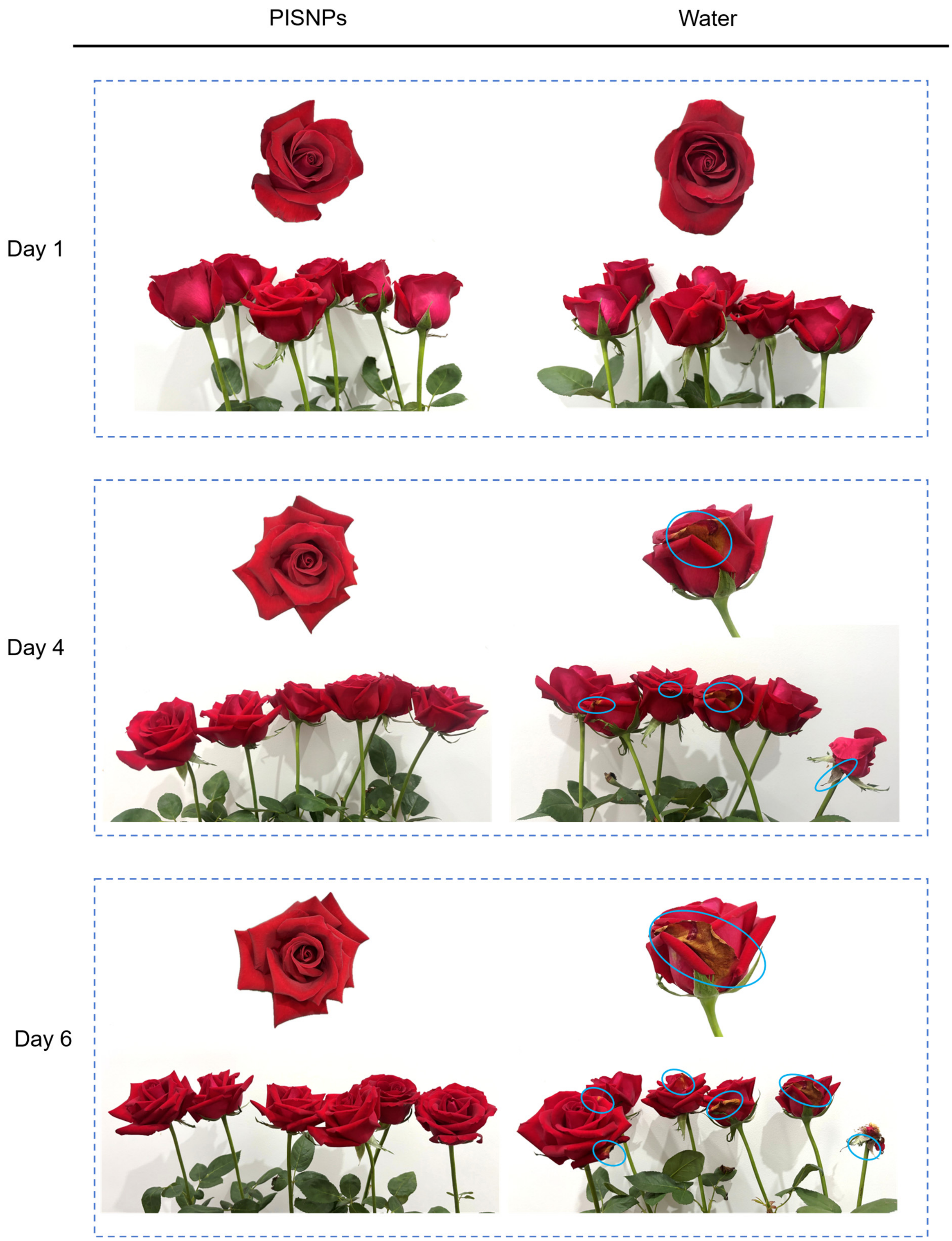

Figure S23. Representative photographs of cut rose treated with PISNPs or deionized water (control).

Table S1 Korsmeyer-Peppas model fitting parameters for PYR release from PISNPs with different polymer concentrations.

Polymer Concentration (mg/mL)	Korsmeyer-Peppas model		
	k (h^{-n})	n	R^2
0.02	0.25	0.45	0.97
0.05	0.28	0.41	0.96
0.1	0.21	0.45	0.99
0.2	0.11	0.67	0.99
0.5	0.05	0.93	0.99
1	0.05	0.84	0.99

Table S2. Korsmeyer-Peppas model fitting parameters for PYR release from PISNPs with different PDMA block ratios.

PDMA Ratio (wt%)	Korsmeyer-Peppas model		
	k (h^{-n})	n	R^2
0	0.19	0.47	0.99
25	0.12	0.55	0.99
50	0.13	0.50	0.99
75	0.16	0.45	0.98
100	0.17	0.48	0.99
